# Learning a Second Language in Adulthood Changes Subcortical Neural Encoding

**DOI:** 10.1155/2020/8836161

**Published:** 2020-10-20

**Authors:** Dongxin Liu, Shuo Wang, Qi Gao, Ruijuan Dong, Xinxing Fu, Esther Pugh, Jiong Hu

**Affiliations:** ^1^Otolaryngology—Head & Neck Surgery, Beijing Tongren Hospital, Beijing Institute of Otolaryngology, Capital Medical University, Beijing, China; ^2^House Ear Institute, 2100 W 3rd St., Los Angeles, CA, USA; ^3^Department of Audiology, University of the Pacific, San Francisco, CA, USA

## Abstract

Second language learning has been shown to impact and reshape the central nervous system, anatomically and functionally. Most of the studies on second language learning and neuroplasticity have been focused on cortical areas, whereas the subcortical neural encoding mechanism and its relationship with L2 learning have not been examined extensively. The purpose of this study was to utilize frequency-following response (FFR) to examine if and how learning a tonal language in adulthood changes the subcortical neural encoding in hearing adults. Three groups of subjects were recruited: native speakers of Mandarin Chinese (native speakers (NS)), learners of the language (L2 learners), and those with no experience (native speakers of foreign languages (NSFL)). It is hypothesized that differences would exist in FFRs obtained from the three language experience groups. Results revealed that FFRs obtained from L2 learners were found to be more robust than the NSFL group, yet not on a par with the NS group. Such results may suggest that in human adulthood, subcortical neural encoding ability may be trainable with the acquisition of a new language and that neuroplasticity at the brainstem level can indeed be influenced by L2 learning.

## 1. Introduction

There is ample literature on neuroplasticity as it relates to bilingualism and second language (L2) learning [1–5]. Learning two languages simultaneously is typically defined as simultaneous bilingualism. This often occurs at a younger age when spoken language learning occurs with relative ease (e.g., children growing up in bilingual households) [6, 7]. Bilingualism can also be achieved by learning L2 after forming his or her native language, which is typically defined as sequential bilingualism [7, 8]. Both simultaneous bilingualism and sequential bilingualism have been shown to impact the central and peripheral nervous system, indicating its adaptive and plastic nature. For example, in the simultaneous bilingual population, it has been found that at the cortical level, grey matter density, cortical thickness, and white matter integrity differ from those in the monolingual groups [7]. At the subcortical level, researchers have examined pitch coding capacity in children and adult bilingual subjects who learned an L2 at younger ages (before age 3) and found that subcortical neural activity was also experience-dependent: their brainstem responses were higher in amplitude than their monolingual counterparts [9, 10].

Sequential bilingualism and L2 learning and their relationship to neuroplasticity, especially for those learning a second language in their adulthood, have garnished attention in more recent years [10, 11]. With the help of rapidly advancing technology in neuroimaging and other techniques, researchers have been able to better understand the neuroplasticity of L2 learning in adulthood. For example, it has been found that adults who were immersed in Chinese learning courses demonstrated increased white matter density compared to their monolingual control counterparts [11]. At the subcortical level, however, little research exists regarding how the brainstem changes its encoding of information during or after the L2 learning experience, especially in adulthood.

One of the more recently utilized tools in subcortical neuroplasticity investigations is the scalp-recorded frequency-following response (FFR) [12]. FFR reflects synchronized neural activities at the level of the brainstem, typically elicited by speech stimulus [13]. As a noninvasive far-field potential response, it reflects the synchrony of neural activity generated by the lateral and inferior colliculus in the brainstem [14]. It is believed to reflect the phase-locking activity of multiple generators in the auditory brainstem [15].

FFR has been shown to be an effective tool in assessing the processing of complex sounds along the auditory pathway, especially at the subcortical brainstem level [16–19]. It has been widely utilized in previous studies to evaluate the effects of auditory and music training [20, 21], speech perception in noise [22], and how aging affects the auditory functions [23–26]. Furthermore, the influences of language background on subcortical pitch processing in different groups of people have been studied using FFR. For example, FFRs recorded from Mandarin Chinese (tonal language) speakers and English (nontonal language) speakers have shown that the pitch tracking accuracy and strength, which both reflect subcortical neural coding capacity, were stronger in speakers of Mandarin Chinese than those of English speakers, indicating that the phase-locking ability in the auditory brainstem could be strengthened by long-term language experience [27–29].

Neural coding of the auditory brainstem could be shaped and reshaped by short-term auditory training and long-term musical training, indicating that both auditory training and musical training have effects on the speech processing of the auditory brainstem [19, 21, 31]. Speech-evoked FFRs in newborn infants from different language background families, Mandarin Chinese and American English, have been shown to be very similar to each other, suggesting that the aforementioned difference in adulthood may indeed be the result of long-term language exposure [31]. In other words, neuroplasticity as a product of long-term auditory influence is evident at the subcortical level. However, to date, there have been few studies aimed at how L2 learning in adulthood could change one's subcortical neural encoding, reflected by measurements such as FFR.

The aim of the current study was to investigate the impact of L2 learning and exposure on an adult's subcortical pitch coding ability, measured by speech-evoked FFR. It was hypothesized that adult L2 learners of a tonal language would have more robust speech-evoked FFRs than those who do not have any exposure to the specific language, yet weaker than those obtained from native speakers.

## 2. Material and Methods

### 2.1. Participants

Three groups of subjects were recruited in this study: native speakers (NS), second language learners (L2 learners), and native speakers of foreign languages (NSFL). For the NS group, 15 native speakers of Mandarin Chinese (seven males and eight females, 24.10 ± 3.3 years of age) were recruited from the local community in Beijing, China.

Another 15 participants who were learning Mandarin Chinese were recruited for the L2 learner group (five males and ten females, 23.35 ± 1.27 years of age). These participants were native speakers of Indonesian, which is not a tonal language. Participants in the L2 learner group had no prior exposure to Mandarin Chinese until they started learning (7.39 ± 2.56 years in experience) Mandarin Chinese in Beijing, China. All L2 learners had achieved at least a level five on the Hanyu Shuiping Kaoshi (HSK). HSK is a standardized test of Mandarin Chinese as a foreign language. It focuses on nonnative speakers' ability to use Mandarin Chinese for communication in daily life, study, and work [32]. HSK, which has six levels with level six being the highest, is considered to be one of the most influential language examinations for Mandarin Chinese [32]. A level five HSK is frequently considered the benchmark of effective verbal communication skills in Mandarin Chinese for nonnative speakers.

Another 15 participants (ten males and five females, 22.07 ± 2.21 years of age) were recruited for the NSFL group. None of them had any experience in learning Mandarin Chinese. Of these 15 participants, eight were native speakers of Bengali, five were native speakers of Dutch, and two were native speakers of Hindi, none of which are tonal languages.

None of the 45 participants had any prior history of otological, audiological, neurological, or psychological issues. They also reported no prior training or involvement in music. This was determined by the participants completing a modified version of the Munich Music (MUMU) Questionnaire [33]. The modified MUMU Questionnaire included four questions on whether they had ever trained to play any musical instruments and whether they had received professional musical or vocal training.

All participants provided written consent for this study, which was approved by the Intuitional Review Board at the Beijing Institute of Otolaryngology and Beijing Tongren Hospital.

### 2.2. Audiometry

**Figure 1:**
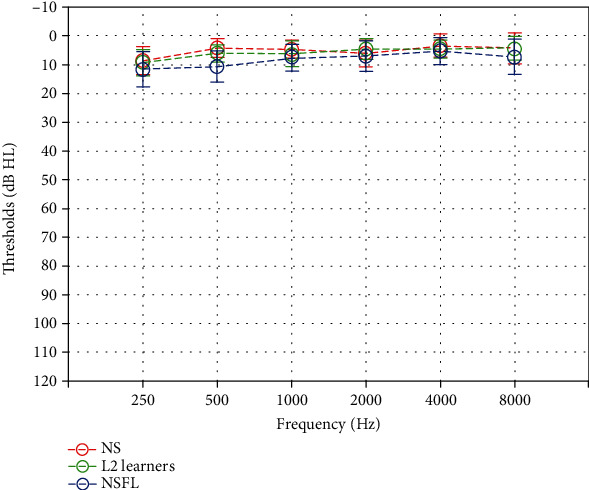
Air conduction pure tone audiograms obtained from all three groups.

Pure tone audiometry, using the cornea two-channel audiometer and insert earphones in a sound treated room, with octave frequencies between 250 Hz and 8000 Hz, revealed hearing thresholds below 15 dB HL in all participants (Figure 1). Click-evoked auditory brainstem responses (ABR) were measured (Intelligent Hearing System, Miami, USA) in all participants with 100 *μ*s rarefaction clicks at 80 dB SPL. Descriptive data of waves I, III, and V of click-evoked ABR is shown in Table 1. No statistically significant group differences were found between any two groups in latencies of wave I, III, or V (Table 2).

### 2.3. FFR Stimuli and Procedures

FFR stimuli used in this study were Chinese syllables /yi/ with rising tone and falling tone, marked as /yi2/ and /yi4/, respectively. They were recorded from a male native speaker of Mandarin Chinese, with an overall duration of 250 ms, including 5 ms each of rising time and falling time. The fundamental frequency (F0) trajectory of the /yi2/ stimulus changed from 120 Hz to 155 Hz and from 180 Hz to 130 Hz for /yi4/. The formant frequencies of the two stimuli were F1 = 400 Hz, F2 = 2100 Hz, F3 = 3000 Hz, and F4 = 3500 Hz. Each stimulus was presented via an electromagnetic shielded ER-3 insert earphone at 70 dB SPL at a rate of 3.2 times per second to the right ear 2000 times.

Participants rested in a supine position with their eyes closed. A typical one-channel electroencephalogram (EEG) recording montage was utilized: gold-plated recording electrodes were placed at the high forehead for the noninverting, right mastoid for the inverting, and low forehead for the ground (all impedances were kept ≤3 k*Ω*). EEG was recorded using the SmartEP system by Intelligent Hearing Systems (Miami, FL, USA) with an internal recording sampling rate of 13,333 Hz (sampling interval of 75 *μ*s). The online band-pass filter was set between 100 Hz and 3000 Hz. Any recording sweep that yielded background EEG amplitudes larger than 25 *μ*V was rejected. Measurements started with click-evoked ABR, followed by FFR stimuli /yi2/ and /yi4/. Another click-evoked ABR was performed at the end of each session to ensure the quality of the EEG recordings.

### 2.4. Data Analysis

EEGs obtained from participants were analyzed to examine the magnitude of the FFR elicited from the stimulus in all three groups. A MATLAB (MathWorks, Natick, MA) pitch tracking script was used for the EEG analysis. An index called Pitch Strength was utilized to quantitatively represent the magnitude of the responses. To calculate the Pitch Strength of each FFR recording, an autocorrelation function was performed on the EEG data resulting in a series of autocorrelation coefficients, which were normalized between 0 and 1. Pitch Strength is defined as the difference between the maximum and minimum autocorrelation coefficients of the EEG signal [28]. It represents the periodicity preserved in the FFR recording, which was a physiological response to the pitch information of the stimulus. Therefore, Pitch Strength, ranging between 0 and 1, demonstrates the preciseness and robustness of the auditory brainstem's ability in accurately following the frequency information in the stimulus, in the case of this study, tonal information in the Mandarin Chinese voice syllables.

To test the hypothesis, an analysis of variance (ANOVA) was performed to compare the effect of language experience (three groups: NS, L2 learners, and NSFL) and tone type conditions (two groups: /yi2/ rising tone and /yi4/ falling tone) on Pitch Strength. A Tukey HSD post hoc test was carried out on the language experience group differences. Statistical level of significance was set at *p* < 0.05.

## 3. Results

### 3.1. Temporal Waveforms and Spectrograms

**Figure 2:**
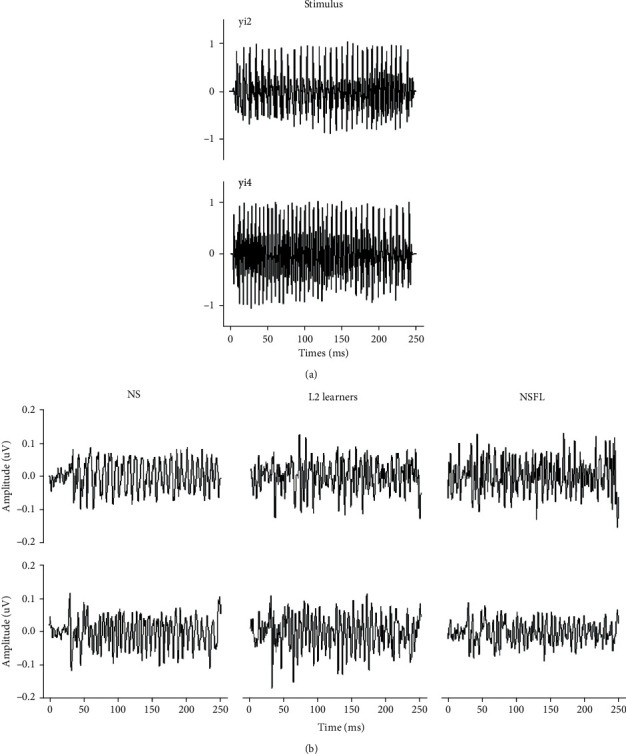
Temporal waveforms of the stimuli (a) and FFR (b) elicited by /yi2/ (top panel) and /yi4/ (bottom panel) from all three participant groups.

**Figure 3:**
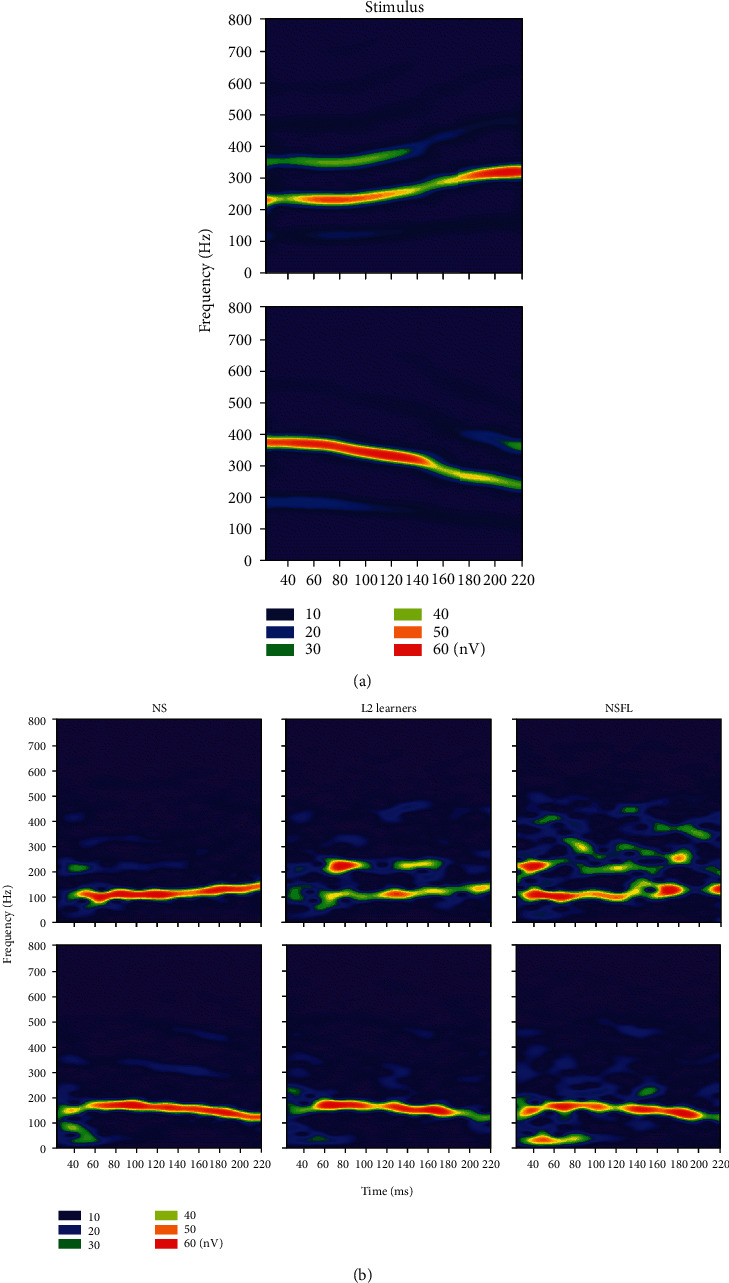
Short-term spectrograms of the stimuli (a) and FFR (b) elicited by /yi2/ (upper panel) and /yi4/ (lower panel) from all three groups. The color warmth scale represents the magnitude of spectral energy in nV. Note the concentrated spectral energy at the F0 range in the FFR recordings.

Temporal waveforms of FFRs elicited by stimuli /yi2/ and /yi4/ were plotted, as shown in Figure 2. Upon visual inspection, it could be observed that the group averaged FFRs from the NS group had clear periodicity from both stimuli, whereas those from L2 learners and NSFL groups were less periodic and more “random.” Similarly, short-time spectrograms based on averaged FFRs in the three groups were also calculated, as shown in Figure 3. Clear, continuous, and robust spectral energy in the FFR obtained from the NS group could be seen, and such spectral energy follows the fundamental frequency (100-200 Hz) of the stimuli. In contrast, spectral energy in the NSFL' FFRs was not as concentrated nor as robust as the NS for both /yi2/ and /yi4/ stimuli, whereas the L2 learners' FFRs were in between.

### 3.2. Statistical Analysis

**Figure 4:**
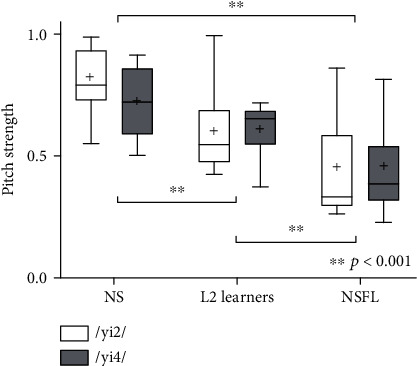
Pitch Strength obtained from all three groups, elicited by stimuli /yi2/ and /yi4/. Boxes represent the interquartile range with whisker bars indicating the ranges. Solid lines represent the median whereas crosses mark the means.

One-way ANOVA with repeated measures tested the effects of language experience and tone type on Pitch Strength. Results revealed a statistically significant difference in Pitch Strength among the three groups with different language experiences (*F*(2, 84) = 30.05, *p* < 0.001). Post hoc comparisons using the Tukey HSD test indicated significantly stronger Pitch Strength in the NS group than the L2 learner group (*p* < 0.001) and the NSFL group (*p* < 0.001). Pitch Strength obtained from the L2 learner group was also significantly stronger than that from the NSFL group (*p* < 0.001) (Figure 4).

No statistically significant difference was found between the two tone types (*F*(1, 84) = 0.622, *p* = 0.433), nor the interaction between language experience and tone type (*F*(2, 84) = 0.982, *p* = 0.379) on Pitch Strength.

## 4. Discussion

The current study compared speech-elicited FFRs obtained from native Mandarin speakers (NS), second language learners who were not native speakers of any tonal languages (L2 Learners), and native speakers of foreign languages who did not have any experience in tonal languages (NSFL). Results showed significant group differences in Pitch Strength, an index representing subcortical pitch processing ability, in these three groups with different language experiences. It suggests that at the brainstem level, neural encoding capacity of certain language-specific acoustic features, in this case, pitch information manifested as tones in Mandarin Chinese, can be influenced by the amount of exposure to and the active processes of learning that specific language, even in adulthood. The difference in subcortical pitch encoding ability between native speakers and native speakers of foreign languages found in this study is consistent with some of the previous studies [27, 28] where native speakers of tonal languages showed more accurate and robust FFRs than those who did not speak any tonal languages.

In addition to confirming experience-dependent neural plasticity at the subcortical level, the current study adds that such subcortical plasticity exists not only in adults who have had life-long language exposure but also in those who are learning a second language in their adulthood. Pitch Strength obtained from L2 learners was shown to be lower than that from the native speakers, but higher than that from the native speakers of foreign languages. This suggests that after several years of learning a tonal language, the L2 learners' auditory brainstem may have “adapted” to better process pitch information. To our knowledge, this is the first time that subcortical neural changes following L2 learning in adulthood is reported in literature.

The underlining mechanism in how auditory exposure or learning experience influences the brainstem's pitch coding ability has been discussed in previous works. For example, a brief auditory training scheme was shown to have elevated pitch coding accuracy in children with learning disabilities, reflected by their enhanced FFR posttraining [20]. One possible explanation provided by the authors was that auditory training may have improved the neural synchronies in the auditory brainstem, which in turn improved the preciseness of neural processing of complex speech sounds. Similarly, more accurate and robust subcortical neural representations of F0 trajectories in lexical tones were obtained in the population of musicians compared with nonmusicians [30]. It was proposed that musician's training has imposed higher demand for precise and efficient subcortical pitch extraction, which resulted in strengthened brainstem responses to the voice pitch.

In the current study, it is proposed that the extensive exposure to tonal information in Mandarin Chinese during L2 learners' learning experience served as a means of auditory training that ultimately leads to the reshaping of their subcortical neural coding ability. It has been proposed that linguistically relevant auditory signals begin to be processed before they reach the cortex. One possible theory discussed in previous studies [27–29] is that the phase-locking activities of neurons may be enhanced by both actively exciting pitch-relevant intervals and/or inhibiting irrelevant intervals. Interspike discharges of these neurons are possibly subject to corticofugal egocentric selection [34, 35]. Learning Mandarin Chinese as a second language, as experienced by the L2 learners in the current study, may have introduced similar corticofugal processes in enhancing neural sensitivity to specific linguistic and acoustic features such as tones. This may result in an enhanced neural phase-locking representation of those tones in Mandarin Chinese at the brainstem level and in turn facilitate better speech and language perception in the cortex.

Results from this study have also provided an interesting observation where FFRs obtained from L2 learners not only reflected a modification in their subcortical pitch coding ability after learning the language for years (7.39 ± 2.56 years); their FFRs also seemed to “fit” right between the native speakers of foreign languages who had no language exposure whatsoever and had the weakest FFR and the native speakers, who clearly had lifelong (24.10 ± 3.3 years) exposure and the most robust responses. It begs the question of after they have started learning a tonal language, at what point does the L2 learners' auditory brainstem start to adapt and reshape to better encode pitch information and to better facilitate tonal information processing. This can only be answered by extending the current study into Mandarin Chinese learners who are less proficient than the current participants and, ideally, follow them through their language acquisition experience. The future direction could also potentially answer the question of if, and when, the subcortical pitch coding capacity of those L2 learners, who are learners of a different language in their adulthood, could ever achieve the same accuracy and robustness of those who are native speakers of that language.

One aspect that we are aiming to improve in the ongoing expansion of the current study is the relatively diverse language backgrounds of the L2 learners and NSFL. Although all L2 learners were native speakers of Indonesian, the NSFL group had more variable language backgrounds: Dutch, Hindi, Bengali, and Indonesian. None of these languages are tonal or utilize pitch in a lexical context, and this variability should not have significantly impacted the results of this study since FFR focuses mainly on pitch coding ability. Nonetheless, differences in acoustic and linguistic features in these languages may have played a subtle role and future studies will try to refine the recruiting process to minimize such impact.

## 5. Conclusions

Results of this study demonstrated that the experience of learning Mandarin Chinese as a second language can enhance the subcortical pitch coding capacity of native speakers of foreign languages, as reflected by the FFR. It is proposed that second language learning experience could potentially reshape the subcortical neural wiring to better facilitate the processing of certain acoustic and linguistic features in a specific language, even in adulthood.

## Figures and Tables

**Table 1:** ABR latencies of waves I, III, and V in three groups.

	NS group			L2 learner group			NSFL group		
	Wave I	Wave III	Wave V	Wave I	Wave III	Wave V	Wave I	Wave III	Wave V
Latencies (ms)	1.56 ± 0.12	3.60 ± 0.10	5.68 ± 0.11	1.57 ± 0.11	3.65 ± 0.12	5.71 ± 0.13	1.56 ± 0.10	3.62 ± 0.13	5.68 ± 0.13

**Table 2:** *t*-test results of ABR latencies among three groups.

	Wave I			Wave III			Wave V		
Group comparison	NS L2 learners	NS NSFL	L2 learners NSFL	NS L2 learners	NS NSFL	L2 learners NSFL	NS L2 learners	NS NSFL	L2 learners NSFL
*t*-test results	*t*_14_ = −0.1893	*t*_14_ = 0.0649	*t*_14_ = 0.1979	*t*_14_ = −0.9457	*t*_14_ = −0.2438	*t*_14_ = 0.6422	*t*_14_ = −0.9763	*t*_14_ = −0.0346	*t*_14_ = 0.8771
	*p* = 0.8526	*p* = 0.9492	*p* = 0.8460	*p* = 0.3603	*p* = 0.8110	*p* = 0.5311	*p* = 0.3455	*p* = 0.9729	*p* = 0.3952

## Data Availability

The TXT data used to support the findings of this study were supplied by Shuo Wang under license and so cannot be made freely available. Requests for access to these data should be made to Shuo Wang, shannonwsh@aliyun.com.

## References

[1] Borsa V. M., Perani D., Della Rosa P. A. (2018). Bilingualism and healthy aging: aging effects and neural maintenance. *Neuropsychologia*.

[2] Burgaleta M., Sanjuán A., Ventura-Campos N., Sebastian-Galles N., Ávila C. (2016). Bilingualism at the core of the brain. Structural differences between bilinguals and monolinguals revealed by subcortical shape analysis. *NeuroImage*.

[3] Costa A., Sebastián-Gallés N. (2014). How does the bilingual experience sculpt the brain?. *Nature Reviews Neuroscience*.

[4] del Maschio N., Sulpizio S., Gallo F., Fedeli D., Weekes B. S., Abutalebi J. (2018). Neuroplasticity across the lifespan and aging effects in bilinguals and monolinguals. *Brain and Cognition*.

[5] Kaiser A., Eppenberger L. S., Smieskova R. (2015). Age of second language acquisition in multilinguals has an impact on gray matter volume in language-associated brain areas. *Frontiers in Psychology*.

[6] Redmond S. (1992). The critical period hypothesis for language acquisition and It's Implication for the management of communication disorders. *National Student Speech Language Hearing Association Journal*.

[7] Li P., Legault J., Litcofsky K. A. (2014). Neuroplasticity as a function of second language learning: anatomical changes in the human brain. *Cortex*.

[8] Legault J., Grant A., Fang S. Y., Li P. (2019). A longitudinal investigation of structural brain changes during second language learning. *Brain and Language*.

[9] Maggu A. R., Zong W., Law V., Wong P. C. M. Learning two tone languages enhances the brainstem encoding of lexical tones.

[10] Skoe E., Burakiewicz E., Figueiredo M., Hardin M. (2017). Basic neural processing of sound in adults is influenced by bilingual experience. *Neuroscience*.

[11] Cao F., Sussman B. L., Rios V. (2017). Different mechanisms in learning different second languages: evidence from English speakers learning Chinese and Spanish. *NeuroImage*.

[12] Moushegian G., Rupert A. L., Stillman R. D. (1973). Laboratory note. Scalp-recorded early responses in man to frequencies in the speech range. *Electroencephalography and Clinical Neurophysiology*.

[13] Smith J. C., Marsh J. T., Brown W. S. (1975). Far-field recorded frequency-following responses: evidence for the locus of brainstem sources. *Electroencephalography and Clinical Neurophysiology*.

[14] Møller A. (1998). Neural generators of the brainstem auditory evoked potentials. *Seminars in Hearing*.

[15] Krizman J., Kraus N. (2019). Analyzing the FFR: a tutorial for decoding the richness of auditory function. *Hearing Research*.

[16] Aiken S. J., Picton T. W. (2008). Envelope and spectral frequency-following responses to vowel sounds. *Hearing Research*.

[17] Cunningham J., Nicol T., Zecker S., Bradlow A. R., Kraus N. (2001). Neurobiologic responses to speech in noise in children with learning problems: deficits and strategies for improvement. *Clinical Neurophysiology*.

[18] Skoe E., Kraus N. (2010). Auditory brain stem response to complex sounds: a tutorial. *Ear and Hearing*.

[19] White-Schwoch T., Carr K. W., Anderson S., Strait D. L., Kraus N. (2013). Older adults benefit from music training early in life: biological evidence for long-term training-driven plasticity. *Journal of Neuroscience*.

[20] Russo N. M., Nicol T. G., Zecker S. G., Hayes E. A., Kraus N. (2005). Auditory training improves neural timing in the human brainstem. *Behavioural Brain Research*.

[21] Bidelman G. M., Alain C. (2015). Musical training orchestrates coordinated neuroplasticity in auditory brainstem and cortex to counteract age-related declines in categorical vowel perception. *The Journal of Neuroscience*.

[22] Hornickel J., Skoe E., Nicol T., Zecker S., Kraus N. (2009). Subcortical differentiation of stop consonants relates to reading and speech-in-noise perception. *Proceedings of the National Academy of Sciences of the United States of America*.

[23] Clinard C. G., Tremblay K. L., Krishnan A. R. (2010). Aging alters the perception and physiological representation of frequency: evidence from human frequency-following response recordings. *Hearing Research*.

[24] Clinard C. G., Cotter C. M. (2015). Neural representation of dynamic frequency is degraded in older adults. *Hearing Research*.

[25] Wang S., Hu J., Dong R. (2016). Voice pitch elicited frequency following response in Chinese Elderlies. *Frontiers in Aging Neuroscience*.

[26] Liu D. X., Hu J., Dong R., Chen J., Musacchia G., Wang S. (2018). Effects of inter-stimulus interval on Speech-Evoked Frequency-Following Response in elderly adults. *Frontiers in Aging Neuroscience*.

[27] Krishnan A. (2002). Human frequency-following responses: representation of steady-state synthetic vowels. *Hearing Research*.

[28] Krishnan A., Xu Y., Gandour J. T., Cariani P. (2004). Human frequency-following response: representation of pitch contours in Chinese tones. *Hearing Research*.

[29] Krishnan A., Xu Y., Gandour J., Cariani P. (2005). Encoding of pitch in the human brainstem is sensitive to language experience. *Cognitive Brain Research*.

[30] Wong P. C. M., Skoe E., Russo-Posaran N., Dees T. (2007). Musical experience shapes human brainstem encoding of linguistic pitch patterns. *Nature Neuroscience*.

[31] Tsao F. M. (2017). Perceptual improvement of lexical tones in infants: effects of tone language experience. *Frontiers in Psychology*.

[32] Zhang J. J. (2013). My opinions on the construction of Hanyu Shuiping Kaoshi (HSK) bank. *Measurement and Assessment*.

[33] Frederigue-Lopes B., Natália M. C. B., Costa O. A. (2015). Munich Music Questionnaire: adaptation into Brazilian Portuguese and application in cochlear implant users. *CoDAS*.

[34] Gao E., Suga N. (2000). Experience-dependent plasticity in the auditory cortex and the inferior colliculus of bats: role of the corticofugal system. *Proceedings of the National Academy of Sciences*.

[35] Suga N., Gao E., Zhang Y., Ma X., Olsen J. F. (2000). The corticofugal system for hearing: recent progress. *Proceedings of the National Academy of Sciences*.

